# ATOUM 6: does a multimodal intervention involving nurses reduce the use of antibiotics in French nursing homes? A protocol for a cluster randomized study

**DOI:** 10.1097/MD.0000000000014734

**Published:** 2019-03-15

**Authors:** Mathieu Ahouah, Pierre Lombrail, Gaétan Gavazzi, Taghrid Chaaban, Monique Rothan-Tondeur

**Affiliations:** a Nursing Research Chair, Laboratory Educations and Health Practices, University Paris 13; b Laboratory Educations and Health Practices, Department of Public Health, University Paris 13, Avicenne Hospital, Bobigny; c University Clinics of Geriatrics, University Hospital of Grenoble-Alpes, GREPI EA7408 University of Grenoble Alpes, Grenoble; d Assistance Publique des hopitaux de Paris (APHP), France.

**Keywords:** antibiotic, communication, nursing homes, participation

## Abstract

**Introduction:**

Urinary tract infection (UTI) is common in elderly living in nursing homes, and antibiotics prescription for this infection is particularly challenging. In these facilities, due to the absence of on-site physicians, nurses play an essential role when an infection is suspected, as they are the ones who collect and communicate by phone all the information needed by the physician for the decision-making process. In that context, our study aims to reduce antibiotic consumption in nursing homes, using a multimodal intervention, by strengthening nurses’ involvement during the process of prescription for UTI.

**Methods/design:**

This is a planned 2-arm cluster randomized study of 40 nursing homes randomly assigned either to the control group or to the intervention group, using a 1:1 ratio. The intervention consists of reinforcing the nurses’ knowledge concerning antibiotics and UTI; assist their clinical judgment using a decision aid diagram; improving their communication skills with the residents, their relatives, and the prescribers; and also increasing their involvement in the intervention’ process by organizing a competition opposing the nursing homes of the interventional group to select additional intervention tools.

**Analysis:**

The main outcome is the reduction of the relative frequency of antibiotics prescription for UTIs in the interventional group.

**Ethics and dissemination:**

Ethics approval was obtained from the French Committee for the Protection of Persons (N. 19.01.04/SI CNRIPH 18.12.07.48123). An article including the main outcome will be submitted to a peer review journal.

## 1 Introduction

World population is ageing, and this phenomenon is constantly increasing.^[1]^ In France, the proportion of elderly persons is projected to reach one-third by 2050, according to the National Institute of Statistics and Economic Studies. This demographic change will be associated with an increase in the burden of diseases^[2]^ such as physical or cognitive impairment in elderly individuals. In France, at the end of 2015, 728,000 people were attending or living in nursing homes, accounting for 10% of the population aged 75 or over and one-third of those aged 90 or over.^[3]^ In these institutions, infections are common and urinary tract infection (UTI) is among 1 of the most frequent bacterial infections.^[4,5]^ In the past decades, antibiotics use has considerably reduced mortality from infections worldwide, and antibiotics are therefore the main drugs used to fight against bacterial infections in nursing homes. However, their increased, and also their inappropriate use raise major concerns that require urgent implementation of therapeutic strategies for a better use. This overuse or misuse has indeed been associated with the phenomenon of bacterial resistance.^[6]^ Furthermore, polypathology^[7]^ in disable residents of nursing homes leads to polymedication, whose management is challenging,^[8,9]^ as it often contributes to increase the use of medication such as antibiotics. Additionally, physiological changes due to ageing affect the elimination of drugs, which may further expose the individuals to drug-related event.^[10]^ In the process of antibiotics prescription in nursing homes, various stakeholders are involved at different stages, playing different roles.^[11]^

### 1.1 The specificities of the elderly and their antibiotic consumption in French nursing homes

Antibiotics consumption in France remains 1 of the most important in Europe,^[12]^ despite intensive public health campaigns aiming at reducing it, and the elderly are amongst the subpopulations with the highest daily consumption.^[13]^ In France, antibiotic-resistant bacteria accounts for 12,500 deaths per year.^[14]^

Residents living in nursing homes^[8,9]^ are particularly prone to polypathology and infections. The clinical symptoms of UTI are most often atypical and nonspecific in these people. Therefore, the diagnosis is a real challenge for healthcare professionals and may be lead to inappropriate antibiotics prescriptions. Inappropriate use of antibiotics contributes also to an increase of health expenditure.^[15]^ Therefore, taking concrete actions to reduce the economic and human burden of antimicrobial resistance is a public health emergency.^[16]^ The implementation of these actions in nursing homes requires appropriate and targeted strategies in which nurses play a major role.

### 1.2 The role of nurses in the antibiotic prescription process

Nurses’ role in nursing homes in the antibiotics prescribing process is of major importance,^[17,18]^ for various reasons, including the lack of on-site doctors.^[4]^ First, nurses are healthcare professionals with clinical and therapeutic knowledge. They are present daily on site and are constantly interacting with residents, their families, and other health professionals including physicians.

Second, nurses provide daily care to encourage residents to perform daily activities to promote their independence.^[19]^ In addition, nurses are responsible for the comfort needs and ongoing supervision of residents in close collaboration with other health caregivers. As a result, quality of care is closely linked to nursing home staffing, and nurses are the first point of contact for prescribers when infection is suspected. In France, the law allows nurses, but only in emergencies, to take certain necessary measures, including certain prescriptions to improve patients’ health while waiting for a doctor.^[20]^ Thus, the practice of nurses in nursing homes with respect to frail residents requires special attention, as they are more likely to have health problems and are at high risk of hospitalization. Nursing practice in this setting requires special clinical skills and autonomy due to the specificities of the residents’ symptoms. Indeed, most of the time, doctors only come to the site at the request of nurses in nursing homes. Therefore, the role of the nurse is essential when an infection requiring a prescription occurs. Physicians’ decisions to examine infected residents in nursing homes are primarily based on their interactions with nurses. It, therefore, seems very relevant, for any strategy related to prescribing and more particularly in the context of antibiotic therapy, to increase nurses’ participation in this process.

### 1.3 Public health intervention strategies aiming at appropriate use of antibiotics in nursing homes

Various actions can be promoted and implemented to ensure the proper use of antibiotics in nursing homes, but certain specificities related to the particular environment of French nursing homes should be taken into account. First, these institutions are characterized by a shortage of nurses and time constraints^[21]^ that are closely linked to workload. Second, strategies aiming at promoting better antibiotic prescription in nursing homes should consider the fact that physicians are most of the time off-site prescribers. Finally, intervention studies on the subject must be designed on the basis of previous evidence-based research. Accordingly, intervention strategies in nursing homes must further promote nurses’ autonomy and be adapted to their specific time constraints. To be effective, an intervention study in this specific context must be designed in a multimodal approach, while carefully considering the characteristics of the prescribing decision-making process, and also the factors that may influence prescription. Such a multimodal intervention could have a real and lasting impact on reducing antibiotic prescriptions.^[22]^

Our study is part of a research program that includes 6 studies. The purpose of the present study is to evaluate the effects of a multimodal intervention involving nurses on reducing antibiotics use for UTI in nursing homes.

## 2 Methods

### 2.1 Design

A 2-arm cluster randomized interventional study is planned to take place in French nursing homes.

### 2.2 Participants/setting

The nursing homes will randomly be selected from the national database of health and social facilities (Table 1). Eligible nursing homes must meet all of the following inclusion criteria: location in Ile-de-France, and presence of nursing staff and registration system available for drug prescriptions. In addition, these facilities should not be part of hospital either, as hospital nursing homes hire on-site physicians who are the prescribers, and as a result, their organization differs from those of facilities with off-site prescribers. In the eligible and selected nursing homes, managers will receive letter that invite their facility to participate. Telephone contact will also be implemented in the recruitment process, and also face-to-face visit in nursing homes.

**Table 1:**
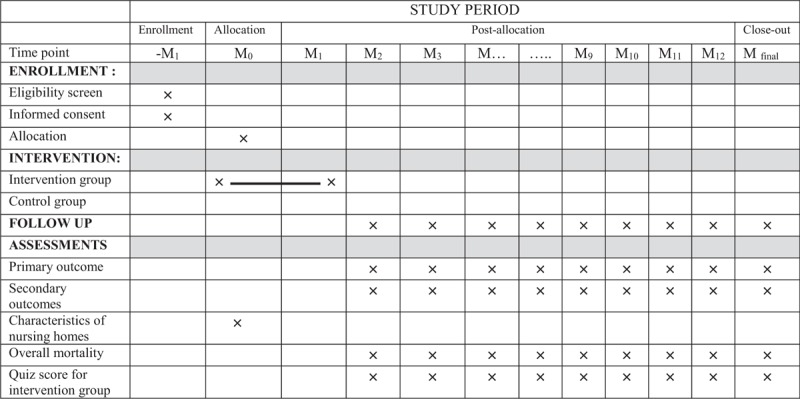
Schedule of enrolment, interventions, and assessments.

### 2.3 Randomization

Eligible nursing homes will be assigned by a 1:1 ratio randomization, either to the intervention or control group, using SAS software. The allocation will be carried out by the clinical research unit of Avicenne Hospital. A new random draw will be carried out whenever a nursing home assigned to the control group will be less than 10 km away from a nursing home assigned to the intervention group, and vice versa, to reduce cross-contamination bias.

### 2.4 Sample size

According to the Enquête nationale de prévalence des infections associées aux soins et des traitements antibiotiques en EHPAD 2016 national survey,^[4]^ the prevalence of antibiotic treatment for UTI was 33.3% in all prescribed systemic antibiotics. In addition, a resident will receive at least 1 prescription of antibiotic on average over a year.^[23]^

**Figure 1:**
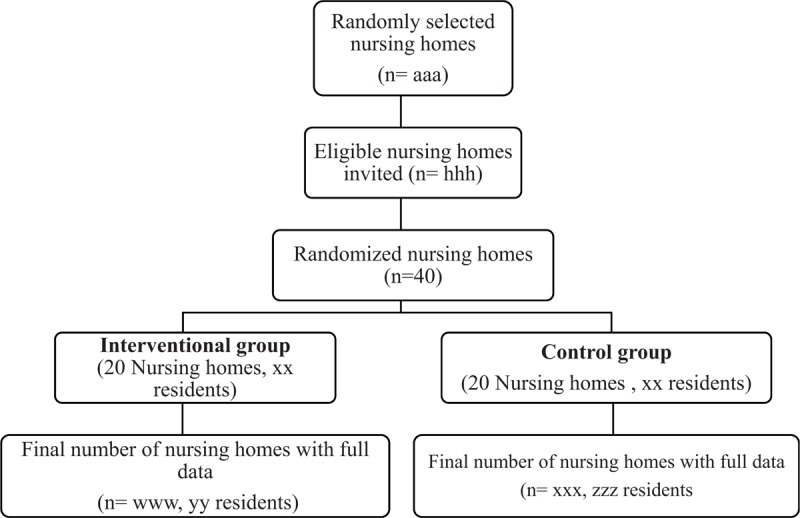
Flow chart of the study.

In our study, we assume that antibiotics for UTI will represent 30% of antibiotics prescriptions for 1 year, and that this percentage will not change in the control group after the intervention. We expect a 20% reduction after 1 year of intervention based on previous data on this issue^[24,25]^ in the intervention group. In a simple randomization, with a power of 80% and an alpha risk of 5%, 124 prescriptions of antibiotics per arm for UTI for a total of 414 prescriptions would be required to show this percentage of decrease in prescriptions for UTI. However, given the clustered design of our study, we must apply an inflation to this value. Additionally, we expect an average of size of 60 residents per nursing home and an intraclass coefficient of 0.05 for an inflation value of 3.95. Accordingly, 15 clusters or nursing homes per arm are required in this study. To anticipate withdrawals of consent, 40 clusters will be included, namely 20 nursing homes in the interventional group and 20 in the control group (Fig. 1).

### 2.5 The intervention

#### 2.5.1 Underpinnings of the intervention

Antibiotics prescribing is a multifactorial process. We have chosen to follow the Predisposing, Reinforcing, and Enabling Constructs in Educational Diagnosis and Evaluation (PRECEDE) phases from the PRECEDE-PROCEED^[26]^ model as the theoretical basis to design the intervention. As part of the PRECEDE phases, we carried out the various diagnoses and evaluations required by this model which stands for Predisposing, Reinforcing, and Enabling Constructs in Educational/ environmental Diagnosis and Evaluation.^[26]^ An unpublished ethnographic study conducted by our research team found that nurses’ communication skills were low, despite their essential role in describing symptoms to physicians by telephone, most often during episodes of suspected UTI. This nurse–prescriber communication in the event of antibiotic prescriptions for UTI is therefore essential because occurring in an environment where there are no doctors on site. This study also highlighted the difficulty nurses have in assessing suspected UTIs in nursing homes due to nonspecific clinical symptoms. Another conclusion of this study is the growing need for nurses to strengthen their knowledge of antibiotic therapy. Thus, the observations of our study on nurses’ communication and clinical judgment are in line with the national roadmap^[14]^ on antibiotics in France. Therefore, the current intervention will focus on nurses’ knowledge as a predisposing factor. Additionally, to assist nurses in their clinical judgment and reasoning, and in their telephone interaction with prescribers, we plan to provide nurses with a decision support diagram and a communication tool. This is the enabling factors of the intervention. Finally, the distribution of posters and pamphlets during visits to nursing homes to meet participants and the organization of a competition targeting the interventional group will be the reinforcing factor. This component aims to support the nurses’ adherence to the intervention, and also the sustainability of the intervention.

#### 2.5.2 Interventional group

**Figure 2:**
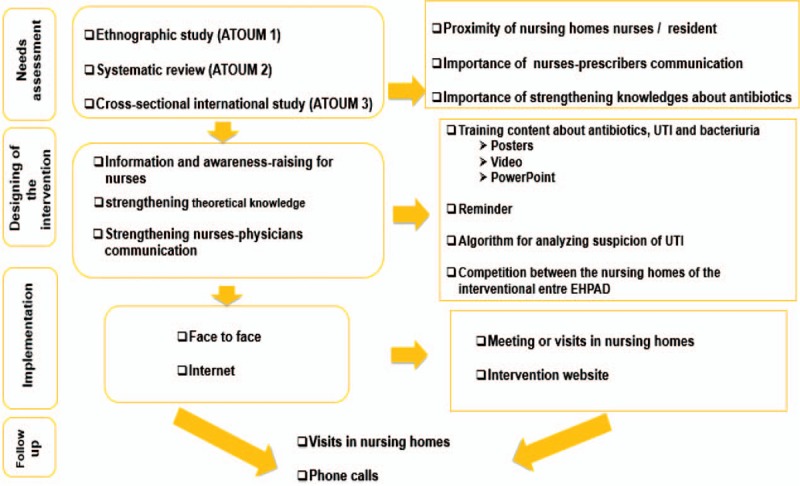
Overview of the intervention.

An interventional group based on 20 nursing homes will benefit a multimodal blended-learning intervention for 1 year. This intervention will include 4 main components, and also 2 implementation modalities. These components will include: awareness and knowledge reinforcement; improvement of communication skills; support for clinical judgment via the use of a decision aid diagram; and improvement of participants’ involvement and the sustainability of the intervention via the organization of a competition. The first implementation modality will take place in the nursing homes with face-to-face tutoring, while the other step will be performed online by self-training (Fig. 2).

At the beginning of the intervention period, investigators will visit each nursing home to meet with the nurses for a 30 minutes face-to-face meeting, to thoroughly describe and explain the intervention. During this meeting, they will also provide intervention tools including web links to access self-training, posters to raise awareness, and the paper version of a diagram. This diagram is a decision aid that first step is a suspicion of UTI by a nurse. Then, she/he will investigate additional symptoms to confirm this first suspicion. The other steps of the diagram include the investigation of the living environment and clinical context of the residents. Finally, after assessing vital signs, nurses will perform a monitoring and decide to call off-site physicians by phone.

The diagram combined to the SBAR tool (Situation, Background, Assessment, and Recommendation) is intended to help nurses analyse suspected UTI and improve phone discussions with off-site prescribers. SBAR is an easy-to-remember technique that allows consistent and structured communication between members of the healthcare team during a critical situation.^[27]^ As part of the follow-up, the first investigator of the paper will return to these institutions every 2 months to renew the posters and reminders, and discuss any problems that participants may have encountered during the online self-training.

For nurses, the internet-based training will consist of consolidating the interprofessional communication with prescribers, communication with residents and relatives, and reinforcing their knowledge. The different topics of this training will cover antibiotics, asymptomatic bacteria, and UTIs described in the PowerPoint presentations, and also a 5-minute video. The diagram described above will be 1 of these online tools. Each training topic on antibiotics, UTI, or resistance will be followed by an online self-evaluation. During this step, video files displaying nurses’ communication situations with physicians and residents or relatives in a UTI episode will be available as basis of the exercises. The marketing and communication department of the national insurance fund will provide technical assistance and support for the recording of these videos.

The last component of the intervention is the organization of a competition. This competition will consist of asking participants, including prescribers and nurses, to design and propose tools such as posters, videos, and slogans aiming at raising awareness on the use of antibiotics, among health professionals in nursing homes.

#### 2.5.3 Follow-up of the interventional group

A lead nurse will be designated as a spokesperson in each interventional nursing home. The research team will follow this group by phone and by visiting the nursing homes regularly during the first 6 months of the intervention period. He will make 1 visit per nursing home every 2 months during the first follow-up period. The last 6 months of follow-up will be done by phone. During the intervention period, 1 member of the research team will call the lead nurses by phone every month to discuss any problems encountered during the online courses, and discuss potential tools or suggestions.

#### 2.5.4 Control group

No intervention is planned for this group, and usual nurses’ practices will take place in these nursing homes.

### 2.6 Outcomes

The main outcome is the reduction in the relative frequency of antibiotic prescriptions for UTI compared with all systemic route prescriptions of antibiotics.

This is defined as the ratio between antibiotics prescriptions for UTI (numerator) and antibiotics prescriptions for any reason (denominator). In this study, a prescription is defined as an order containing at least 1 systemic route antibiotic.

Secondary outcomes are overall antibiotic therapy prescriptions and antibiotics consumption expressed in daily defined dose (DDD) per 1000 resident-days. In addition, a medico-economic analysis is planned.

### 2.7 Data collection and management and monitoring

Many data are expected (Table 2). Data sources will be 3-fold that are the Caisse National d’Assurance Maladie (CNAM) (French National Health Insurance Fund), the included nursing homes, and the webmaster. The CNAM, which has agreed to carry out these data survey, will provide researchers with global antibiotics prescriptions and the antibiotics consumption in DDD month to month. Data provided by the CNAM will also help cost analysis. Included nursing homes will provide reasons for the antibiotics prescriptions when an antibiotic prescription will be identified in the CNAM database. These nursing homes will also provide once characteristics of their facilities, staff, and residents, and month to month overall mortality. The information related to the use of the intervention website will be collected from this website by the researchers and the webmaster. They will collect: internet connexion time, self-training score per nursing homes, and time devoted by investigators to visit nursing homes during the intervention period.

**Table 2:**
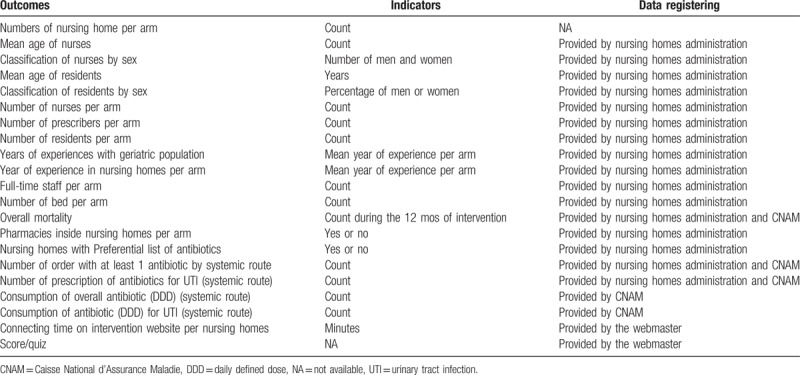
Outcomes expected.

For healthcare staff and residents, data will be collected with nursing home managers or coordination teams (nurses or doctors) during a specific appointment or the first visit. The third author will collect the number of urinalyses performed per nursing home from pharmacies that deliver drugs to nursing homes.

Data management and monitoring will be carried out by a private clinical research team. To ensure quality of data, the researchers have restricted the number of variables to those most relevant according to existing literature. Many numerical and logical controls like possible values for each variable and consistency of information about the same nursing home will be used to ensure data reliability in SAS 9.3.

### 2.8 Statistical analysis

#### 2.8.1 Missing data

Above 5% of missing data, investigators will perform multiple imputations according to the most likely mechanism.

#### 2.8.2 Effect analysis

The main analysis will be performed in intent to treat, and a per-protocol analysis will also be carried out using SAS version 9.3 software with a 5% significance threshold. A mixed model which is the appropriate method in cluster study will be used to assess the impact of the intervention. No interim or subgroup analysis is planned in this study. A Poisson regression with random effects will be carried out to assess the impact of the intervention using secondary endpoints which are the total number of antibiotics per arm and mixed model for the antibiotics consumption expressed in DDD consumed during the year of follow-up of the interventional group. All models will consider the potential confounding factors such as average number of beds, average number of physicians, and average number of comorbidities.

### 2.9 Cost analysis

We will carry out a budget impact^[28]^ assessment because the costs are charged to nursing homes. The cost of the intervention will be estimated by considering the time dedicated by trainers and nurses to the intervention and the material resources used to implement and monitor this intervention. Staff time related to this intervention will be estimated from gross salaries and material resources at the purchase price (with depreciation and maintenance if necessary). Costs will be estimated by comparing antibiotics consumption between the 2 groups and valuing them at the purchase price. The budget impact at 3 and 5 years will be estimated with different scenarios of the intervention, and assumptions on the incidence of UTIs in nursing homes.

## 3 Discussion

The atypical and nonspecific clinical symptoms of UTI in the elderly make its diagnosis challenging in nursing homes and lead to inappropriate antibiotics prescriptions.^[29]^ This consumption of antibiotics is associated with bacterial resistance, which remains a global health problem in France.^[30]^ Therefore, actions to reduce antibiotic consumption in the field of human health are essential to decrease its effect.^[31]^ Nurses are potential actors in achieving this goal in nursing homes due to their important role of symptoms assessment and description to off-site physicians in these facilities. The complexity of antibiotic prescribing involves the implementation of multicomponent interventions that are more effective than simple strategies.^[32]^

Interventions in nursing homes include many organizational challenges. But, despite the positive effects of this approach on reducing drugs use in institutions for elderly people, some studies have shown that the effects decrease over time.^[33]^ Consequently, this kind of interventions requires regular repetition.^[33,34]^ Another potential issue in nursing homes appears to be the methods of delivering of this intervention. In fact, time constraints in face-to-face meetings combined with the financial cost of interventions may explain why positive effects are difficult to maintain. Accordingly, the present study will consider these important aspects and challenges related to the implementation of the interventions in these facilities. For this reason, this intervention will first take into account accessibility. One way of disseminating information could be through the internet to meet the time and schedules constraints of professionals.^[35]^ Indeed, in the digital era, the internet-based interventions represent a better way to perform an intervention because they can reduce waste of time on face-to-face interventions.^[35]^ Nowadays, the internet is a mean and an opportunity to set up trainings suitable to the schedules and workload of professionals. One benefit of internet-based interventions is that they can be less expensive and time-consuming than face-to-face meetings, while allowing discussions in forums.^[36]^ Internet-based interventions promote maintenance by facilitating the training of nurses due to their high turnover in geriatric facilities. However, researchers must check this feasibility before a large-scale intervention. Another challenge of this intervention is its sustainability. In this case, 1 solution consists of involving^[37]^ participants of this study in the design of part or all of the intervention tools such as video and posters to empower participants and facilitate their ownership.

Another point of discussion in this study is its cluster randomized design. The choice of this design is motivated by the challenge of avoiding shared information by nurses of the training planned in the intervention between the control group and the interventional group.^[38]^ Conversely, some authors recommend the stepped wedge cluster randomized trials instead of simple classic cluster design. Stepped wedge is considered a pragmatic design and reconciles policy makers and interventions planners.^[39]^ Whereas the researcher might take a different view.^[39]^ However, researchers recommend stepped-wedge when there is already enough evidence to support the effectiveness of a planned intervention. Thus, since our type of intervention being the first carried out in France in nursing homes could justify its choice in this study.

## 4 Ethics and dissemination

The study protocol was approved by (CPP Ouest III); a French ethics review committee (N. 19.01.04/SI CNRIPH 18.12.07.48123). Moreover, the managers of the participating nursing homes must give their consents. The study researchers will ensure that nurses of each participating nursing home give their informed consent before randomization. Nursing homes may withdraw from the study at any time and we’ll keep a record of the reasons why they want to leave. These records are highly confidential, so the identity of nursing homes and all data of the study will be replaced by pseudonyms. Data collected from this study will be available to person on request. Regarding the dissemination plan, results from the trial will be communicated to the participating facilities and health professionals by organizing meetings within the facilities. In addition, two communications are planned after one year, one to the National Health Insurance Fund and, the second to conferences on the subject of infectious diseases. In addition, a scientific article is planned after the final results are obtained. According to France clinical research law, all protocol amendments will be communicated by the first author to the sponsor which will transfer it to the ethics review committee.

## 5 Trial status

This protocol is registered on clinicalTrials.gov: NCT03180983. This version number is 1.1 Protocol dated January 23, 2019.

## Acknowledgments

We are grateful to the CNAM who accept to support the research team for the communication tools and accept to collect all the data related to the consumption of antibiotics.

Our acknowledgements to the clinical research unit of Avicenne Hospital and to Marouane BOUBAYA the biostatistician who reviewed the sample size calculation and statistics chapter.

## Author contributions

Mathieu Ahouah and Monique Rothan-Tondeur draft the article.

Mathieu Ahouah and Monique Rothan-Tondeur made the design of the intervention

Professor Pierre Lombrail contributed to the design of the intervention.

All the authors reviewed the manuscript.

**Conceptualization:** Mathieu Ahouah, Pierre Lombrail, Taghrid Chaaban, Monique Rothan-Tondeur.

**Data curation:** Mathieu Ahouah, Monique Rothan-Tondeur.

**Formal analysis:** Mathieu Ahouah.

**Funding acquisition:** Monique Rothan-Tondeur.

**Investigation:** Mathieu Ahouah, Monique Rothan-Tondeur.

**Methodology:** Mathieu Ahouah, Pierre Lombrail, Gaétan Gavazzi, Monique Rothan-Tondeur.

**Project administration:** Mathieu Ahouah, Pierre Lombrail, Monique Rothan-Tondeur.

**Resources:** Monique Rothan-Tondeur.

**Validation:** Mathieu Ahouah, Pierre Lombrail, Gaétan Gavazzi, Taghrid Chaaban, Monique Rothan-Tondeur.

**Visualization:** Mathieu Ahouah, Pierre Lombrail, Gaétan Gavazzi, Taghrid Chaaban, Monique Rothan-Tondeur.

**Writing – original draft:** Mathieu Ahouah, Monique Rothan-Tondeur.

Mathieu AHOUAH orcid: 0000-0002-4241-6536.
